# Plant-based diet and risk of all-cause mortality: a systematic review and meta-analysis

**DOI:** 10.3389/fnut.2024.1481363

**Published:** 2024-10-23

**Authors:** Junwen Tan, Shipeng Zhang, Yanjie Jiang, Jie Li, Chuan Yang

**Affiliations:** ^1^Hospital of Chengdu University of Traditional Chinese Medicine, Chengdu, China; ^2^Nanjing University of Chinese Medicine, Nanjing, China

**Keywords:** plant-based diet, meta-analysis, cancer mortality, CVD mortality, all-cause mortality

## Abstract

**Objective:**

A systematic analysis was conducted to determine the relationship between a plant-based diet and all-cause mortality.

**Methods:**

The PubMed, Embase and Web of Science databases were searched. Two authors selected English documents from the database. Then the other two authors extracted the data and evaluated the Newcastle–Ottawa Scale (NOS). This study adhered to the guidelines of the Preferred Reporting Project (PRISMA) and the PROSPERO Registry protocols. A mixed-effects model combined maximum adjusted estimates, with heterogeneity measured using the I^2^ statistic. The sensitivity analysis validated the analysis’s robustness, while publication bias was assessed.

**Results:**

The results of the meta-analysis of 14 articles revealed that a plant-based diet (PDI) can reduce cancer mortality (RR = 0.88, [95% CI 0.79–0.98], τ^2^: 0.02, I^2^: 84.71%), cardiovascular disease (CVD) mortality (RR = 0.81, [95% CI 0.76–0.86], τ^2^: 0.00, I^2^: 49.25%) and mortality (RR = 0.84, [95% CI 0.79–0.89], τ^2^: 0.01, I^2^: 81.99%) risk. Adherence to a healthy plant-based diet (hPDI) was negatively correlated with cancer mortality (RR = 0.91, [95% CI 0.83–0.99], τ^2^:0.01, I^2^:85.61%), CVD mortality (RR = 0.85, [95% CI 0.77–0.94], τ^2^: 0.02, I^2^: 85.13%) and mortality (RR = 0.85, [95% CI 0.80–0.90], τ^2^: 0.01, I^2^: 89.83%). An unhealthy plant-based diet (uPDI) was positively correlated with CVD mortality (RR = 1.19, [95% CI 1.07–1.32], τ^2^: 0.02, I^2^: 80.03%) and mortality (RR = 1.18, [95% CI 1.09–1.27], τ^2^: 0.01, I^2^: 89.97%) and had a certain correlation with cancer mortality (RR = 1.10, [95% CI 0.97–1.26], τ^2^: 0.03, I^2^: 93.11%). Sensitivity analysis showed no contradictory results.

**Conclusion:**

The hPDI was negatively associated with all-cause mortality, and the uPDI was positively associated with all-cause mortality.

**Systematic review registration:**

https://www.crd.york.ac.uk/PROSPERO/#loginpage.

## Introduction

The 2017 Global Burden of Disease study estimated that unhealthy diets were a leading cause of death worldwide (1). According to the 2019 analysis of the global burden of disease and injury, more than 50% of mortality was attributed to chronic conditions such as cancer and cardiovascular diseases (2). Studies have shown that adherence to a high-quality diet is associated with a reduced risk of inflammation and mortality (3). Dietary modification has been identified as one of the most important strategies for preventing cardiovascular disease (CVD) at the population level (4).

A plant-based diet (PBD) refers to a pattern of eating based on plant-based foods, containing fewer or no animal products and more plant products, such as vegetables, fruits, whole grains, legumes, nuts, and seeds (5). Research has linked plant-based diets to a lower risk of various diseases (6),including an inverse association between the intake of fruits and vegetables within a plant-based diet and the risk of coronary heart disease, stroke, cardiovascular disease, total cancer, and all-cause mortality (7). Multiple studies have also demonstrated that nut intake via a plant-based diet is inversely associated with overall CVD and all-cause mortality (8, 9). A plant-based diet contains soy foods that reduce the risk of cardiovascular disease and improve cardiometabolic health (10, 11), while some studies suggest that certain compounds in a plant-based diet may have potential in treating cancer (12).

A comprehensive review of previous studies on plant-based diets revealed that adherence to such a diet can significantly reduce the risk of CVD (13–15). Furthermore, plant-based diets have been associated with a reduction in overall mortality rates (16–18). At the same time, most studies fail to distinguish between the concepts of a vegetarian diet, plant-based diet, and Mediterranean diet, leading to contradictory results. Therefore, standardizing dietary definitions prior to research is of great value to the accuracy of data analysis in these studies. Despite this, there is currently no meta-analysis available on the relationship between plant-based diets and all-cause mortality. This study was based on the concept of the plant-based diet index proposed by Satija et al. (19). Through an integrated analysis of both domestic and foreign studies on plant-based diets and all-cause mortality, we discussed the correlations among the overall plant-based diet index (PDI), healthy plant-based diet index (hPDI), unhealthy plant-based diet index (uPDI), and all-cause mortality. This study was aimed to provide data supporting the association between a healthy plant-based diet and all-cause mortality.

## Methods

### Study design

We conducted a systematic review and meta-analysis of prospective cohort studies to investigate the associations between plant-based diets and cardiovascular death, cancer-related death, and all-cause mortality. The entire process adhered to the Preferred Reporting Project for Systematic Review and Meta-Analysis (PRISMA) 2020 guidelines (20). This research program has been registered in the International Prospective Register of Systematic Reviews (PROSPERO: Registration No. CRD42024535940) (Supplementary material S1).

### Search strategy

Researches on the association between a plant-based diet and all-cause mortality were searched in PubMed, Web of Science, Embase, and other databases from their establishment to July 1, 2024. The search was limited to publications in English. Both subject words and free words were used for the search. The retrieval strategy included medical subject headings (Mesh) and free-word combination designs related to exposure (“plant-based diet,” “plant,” “diet,” and “vegetarian”) and outcomes (“mortality,” “mortality rate,” “death,” “death rate,” “cardiovascular mortality” and “cancer mortality”). Additionally, a manual search of reference lists for articles on similar topics was conducted to identify potential qualifying studies. The detailed search strategies were provided in Supplementary Table S1.

### Eligibility criteria

The inclusion criteria were as follows: (1) study type: cohort study; (2) they adhered to plant-based eating patterns and were assessed based on the PDI, which refers to those who adhered best on the hPDI and uPDI; and (3) plant-based diets were associated with cardiovascular death, cancer-related death, and all-cause death as outcomes of concern.

The exclusion criteria were as follows: (1) duplicate studies reporting the same cohort; (2) incomplete data leading to the inability to calculate the corresponding odds ratio(OR)/relative risk(RR)/hazard ratio(HR); (3) research focused on populations of patients with specific diseases; (4) nonrelevant exposure outcomes (such as stroke, cerebrovascular accident rates, etc.); and (5) nonrelevant study designs (such as intervention studies, case–control studies, cross-sectional studies, reviews, and meta-analyses).

### Assessment of plant-based dietary patterns

Different studies have different definitions of the plant-based diet index (5, 19). This study adopted the concept of the plant-based diet index proposed by Satija et al. (19) According to this concept, plant-based diet index could be divided into overall plant diet index, healthy plant diet index, and unhealthy plant diet index. The healthy plant diet index emphasizes a greater intake of healthy plant-based foods such as whole grains, vegetables, nuts, legumes, coffee and tea (21), whereas the unhealthy plant diet index focuses on less healthy plant-based food groups, including fruit juices, sugary drinks, refined grains, potatoes and sweets/desserts, as well as animal foods such as animal fats, dairy product eggs, fish or seafood red meat and other animal foods (19). Positive scoring is applied to healthy plant foods, whereas reverse scoring is applied to animal foods and less healthy plant foods. The final score for all the components is added to obtain the total PDI score. A higher PDI score indicates better dietary quality. As one of the evaluation index for a plant-based diet, it considers both the quality of consumed plants and the intake of animal food. The application of this evaluation index in the study of plant intake and mortality can be used to comprehensively evaluate the impact of a plant diet on all-cause mortality.

### Study selection

The literature screening process consisted of two steps. Initially, two authors (TJW and ZSP) conducted a thorough search for relevant literature. All identified literature was imported into EndNote X9 software, and any duplicate studies were removed both automatically and manually. Eligible studies were then identified by reviewing the title and abstract according to preestablished inclusion and exclusion criteria. The second step involved a full-text screening of uncertain studies to ultimately decide which ones would be suitable for inclusion in the meta-analysis. In cases of disagreement during the literature selection process, discussions were held with the third author (JYJ) until a consensus was reached. See the attachment for the specific screening process.

### Data extraction and quality assessment

The first author utilized standardized data collection tables to extract pertinent information from eligible studies, whereas the secondary author independently verified these data against the original article. Specifically, the extracted information included the last name and year of publication of the primary author, study name (if applicable), geographical location, study duration and mean follow-up years, specific study design, demographic characteristics (mean age, percentage of women, and BMI), sample size, number of cases and determination of exposure outcomes (cardiovascular deaths, cancer-related deaths, and all-cause mortality), dietary assessment methods, comparisons of dietary characteristics, most fully adjusted risk ratios, adjusted confounders in statistical models, and risk diseases among confounders. The final data extraction was based on a consensus between TJW and ZSP. The quality of the initially included studies was independently assessed via the Newcastle–Ottawa Scale (NOS). The NOS consists of eight items in three dimensions (selection, comparability, and outcome), with a maximum score of nine points for each study: four points for selection criteria, two points for comparability criteria, and three points for outcome evaluation. Studies with scores ranging from 0–3, 4–6, and 7–9 are considered low quality, medium quality, and high quality, respectively. See the annex for the specific NOS rating in Supplementary Table S2.

### Data synthesis and analysis

To investigate the associations between a plant-based diet and cardiovascular death, cancer-related death, and all-cause mortality, we conducted a meta-analysis of multivariate adjusted RR or OR from each study via Stata 16. HR were considered equivalent to RR. We also calculated aggregated risk estimates for the highest and lowest adherence to plant-based dietary patterns (22). Heterogeneity among studies was assessed via I^2^ statistics and visually represented with forest plots. A value of I^2^ ≤ 50% indicated significant heterogeneity, whereas I^2^ > 50% indicated insignificant heterogeneity. If significant heterogeneity was present, we used a random effects model; otherwise, a fixed effect model was employed. To assess the significance of RR differences and potential residual confounding factors, we performed a “leave one out” sensitivity analysis by iteratively removing one study at a time to evaluate its impact on the overall effect estimate. Additionally, we utilized Egger’s test to examine potential publication bias in studies with ten or more included publications and to determine its influence on the overall results.

## Results

**Figure 1:**
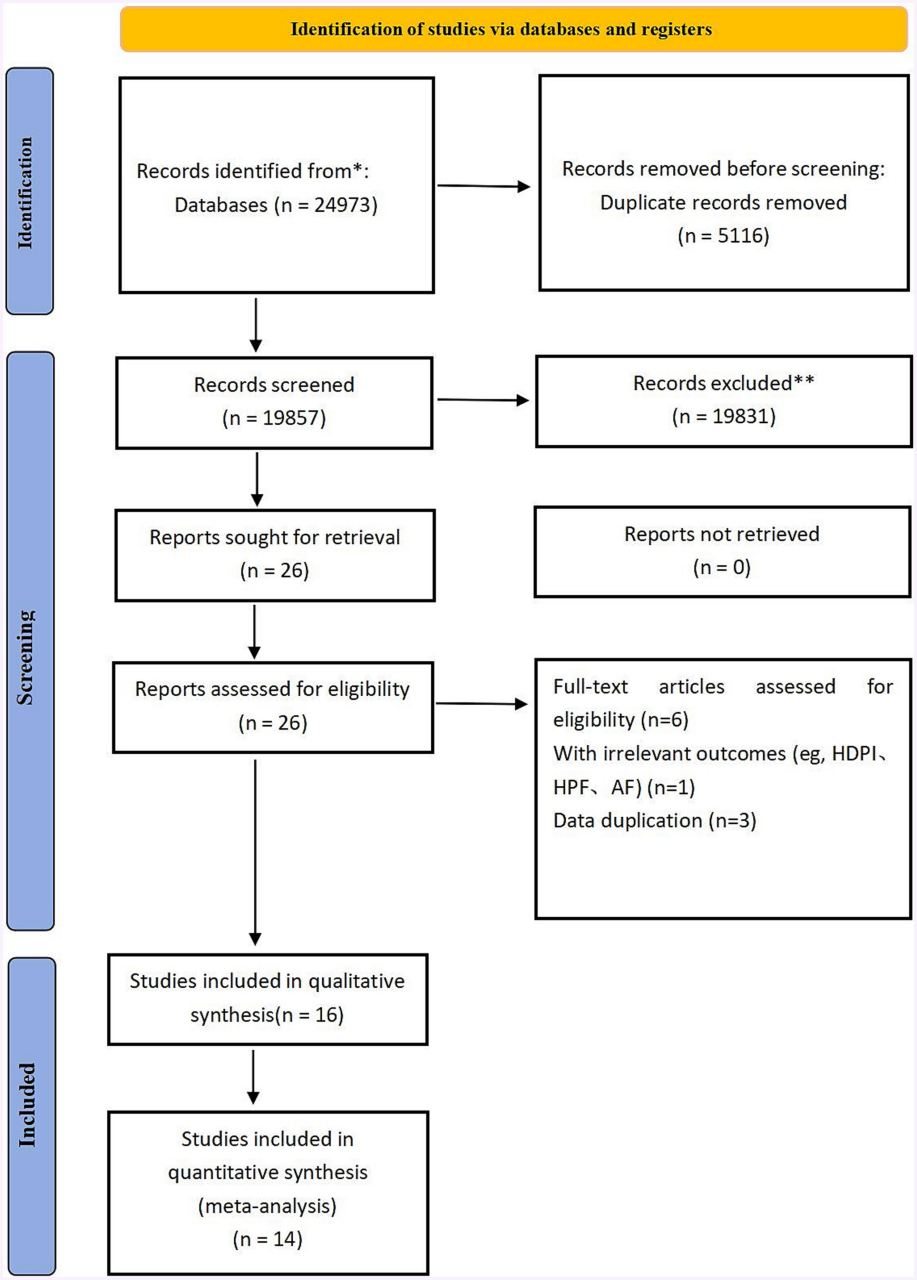
Figure shows the flow chart. It mainly reflects the process of literature retrieval and exclusion.

Table 1 presents the characteristics of the eligible studies; A total of 14 articles with NOS scores greater than 7 were included in the analysis, two of which provided multiple risk estimates due to stratification. The 14 articles collectively involved 976,396 participants, with sample sizes ranging from 1,404 to 315,919 in the included studies (Figure 1). Seven articles focused on cancer-related deaths (1, 16, 23–27), eight articles focused on cardiovascular death (1, 16, 23, 24, 26–29), and fourteen articles focused on all-cause deaths (1, 16, 23–33). A systematic review was conducted to analyse the correlations between the PDI, hPDI and uPDI and mortality-related outcomes. The summary results indicated that adherence to a high level of a healthy plant-based diet was associated with lower mortality, whereas adherence to an unhealthy plant-based diet was associated with higher mortality. Some results showed significant heterogeneity, and subgroup analysis suggested that geographic region, type of BMI classification of the plant-based diet and follow-up interval may be sources of heterogeneity between studies (Table 2).

**Table 1:** Basic research characteristics.

Author, year	Data from	Study type	Time	Age	Female (%)	Case	Total	Bmi	Follow-up time	Dietary assessment mode	Diet type	Outcome	Adjust
Dong D Wang, 2022	MVP	Cohort study	2011–2018	65.5	8.1	31,316	315,919	/	4 year	SFFQ	PDI hPDI uPDI	All mortality, CVD mortality, cancer mortality, other mortality	Age (years: < 60, 60–70, > 70) and sex (male or female); race/ethnicity (non-Hispanic European American, African American or other), education level (≤ high school or GED, some colleague, or college or above), income level (< $30,000, $30,000–$59,000 or ≥ $60000)and marriage status (currently married or not), smoking status (current, former or never smoking), frequency of alcohol consumption (never, < 1 times/week or ≥ 1 times/week), frequency of exercise vigorously (never/rarely, 1–4 times/month,2–4 times/week or ≥ 5 times/week), total energy intake (in quintiles) and BMI (< 23.0, 23.0–24.9, 25.0–29.9, 30.0–34.9 or ≥ 35.0 kg/m2).histories of diabetes, hypertension, hypercholesterolemia, cancer and CVD at baseline (yes vs. no).
Hairong Li, 2022	The US National Health and Nutrition Examination Survey (NHANES).	Cohort study	1999–2014	47.3	/	4,904	40,074	/	7.8 year	24-h dietary assessment	PDI hPDI uPDI	All mortality, CVD mortality, cancer mortality	Sex (male, female), age (spline variables in the analyses with three knots), and total energy intake (spline variables in the analyses with three knots); race/ethnicity (non-Hispanic white, non-Hispanic black, Hispanic or other race), education (≤12th grade, high school graduate/GED or equivalent, or more than high school), marital status (married, widowed/divorced/separated, or never married), ratio of family income to poverty (<1.30, 1.30–3.49, or ≥ 3.50), physical activity (<8.3, 8.3–16.7, or > 16.7 METS h/week), smoking (never smokers, former smokers, or current smokers), drinking (never drinking, low to moderate drinking, heavy drinking), body mass index (<18.5, 18.5–24.9, 25.0–29.9, and ≥ 30.0), diabetes (no, yes), hypertension (no, yes), other CVDs (no, yes), and cancer (no, yes)
Hui Chen, 2022	CLHLS	Cohort study	2008–2018	86.9	57.4	8,937	13,154	20.3	5.7 year	FFQ	PDI hPDI uPDI	All mortality	Age (years), sex (male or female), ethnicity (Han or non-Han), residential area (urban or rural), marital status (married, not married or bereaved), household income (low, medium or high), education (years), smoking status (former smoker, current smoker or never smoker), alcohol intake (former drinker, current drinker or never drinker), regular exercise (yes or no) and baseline BMI (kg m^−2^).
Hyunju Kim, 2019	ARIC study Coordinating Center	Cohort study	1987–2016	53.8	55.2	5,436	12,168	/	25 year	The 66‐item semi-quantitative Willett food frequency questionnaire	PDI hPDI uPDI	All mortality, CVD mortality	Age, sex, race‐center, total energy intake, education, smoking status, physical activity, alcohol consumption, and margarine consumption
Ijeamaka C. Anyene, 2021	The Pathways Study	Cohort study	2005–2013	60	100%	653	3,646	28	9.51 year	Block 2005 Food Frequency Questionnaire (FFQ)	PDI hPDI uPDI	All mortality, breast-cancer-specific mortality	Age at diagnosis, total energy intake (kcal/d), and physical activity (moderate-vigorous MET-hours/week); race/ethnicity, education, menopausal status, smoking status, and stratified by tumor stage and ER status
Ilka Ratjen, 2021	The Biobank popgen	Cohort study	2004–2016	69	44%	204	1,404	26.2	7 year	FFQ	PDI hPDI uPDI	All mortality	Sex, age at diet assessment, BMI, physical activity, survival time from CRC diagnosis until diet assessment, tumor location, metastases, other cancer, type of therapy, smoking status, alcohol intake, total energy intake, time × age, time × BMI, and time × metastases.
Jihye Kim, 2021	A population-based cohort in the Korean Genome and Epidemiology Study_Health Examinees	Cohort study	2004–2019	52.7	65%	3,074	118,577	23.9	/	Semi-quantitative food frequency questionnaire (FFQ)	PDI hPDI uPDI	All mortality, CVD mortality, cancer mortality	Age (continuous), sex (men/women), education (≤6, 7e12, >12 years), smoking status (never/former/current), alcohol consumption (never/former/current), energy intake (continuous), physical activity (yes/no), body mass index (continuous), and disease history (yes/no).
Leah J Weston, 2022	Jackson Heart Study (JHS)	Cohort study	2000–2018	53.8	64	597	3,635	31.75	15 year	Delta NIRI FFQ	PDI hPDI uPDI	All mortality	Age, sex, and total energy intake. educational attainment, smoking status, alcohol intake, margarine intake, and physical activity, body mass index, total cholesterol, hypertension, diabetes, estimated glomerular filtration rate, hormone replacement therapy medication use, and statin medication use.
Lihui Zhou, 2024	UK Biobank	Cohort study	2006–2010	55.99	54.30%	9,335	189,003	26.87	9.6 year	The Oxford WebQ dietary questionnaire	PDI hPDI uPDI	All mortality, Cardiovascular (I), Ischemic heart disease (I20–I25) cancer mortality, Neoplasm (C00-C49), Hematopoietic (C80-97), Respiratory (J), Neurological (G), Mortality	Age, menopausal status or hormone replacement use in females, ethnicity, education, and quintiles of the Townsend deprivation index, familial history of diseases (CVD, diabetes or cancers); smoking status, alcohol drinking frequency, body mass index, physical activity, sedentary time, total dietary energy intake, multivitamin supplement use, and the Charlson Comorbidity Index with the lowest quintiles as references
M Delgado-Velandia, 2022	ENRICA, the National Death Index of Spain	Cohort study	2008–2020	47	54.50%	699	11,825	26.94	10.9 year for Mortality 9.8 year for CVD mortality	Electronic diet history (HD-ENRICA)	hPDI uPDI	All mortality, CVD mortality	Age (years), sex (men, women), and education (≤primary, secondary, and university), smoking status (never, former, and current smoker), body mass index (kg/m^2^), energy intake (kcal/day), alcohol intake (never drinker, former drinker, moderate alcohol intake, excessive alcohol intake), recreational physical activity (tertiles), number of chronic diseases (continuous), and number of medications taken (continuous).
Qian Wang, 2023	At the Shengjing Hospital of China Medical University	Cohort study	2018–2021	61.1	30.64	240	408	22.7	40.97 months	Semi-quantitative food frequency questionnaire (FFQ)	PDI hPDI uPDI	All mortality	Adjusted for age at diagnosis (≥60, <60 years), sex (male, female) and total energy intake (continuous, kcal/d). Additionally adjusted for race(Han, others), region (city, rural), education (junior secondary school or below, senior high school/technical secondary school, junior college/university or above), income per month (<5,000, 5,000–10,000, ≥10,000 RMB), marital status (married, unmarried/divorced/widowed), smoking status (yes, no), alcohol intake (continuous, g/d), diet change (yes, no), BMI (continuous, kg/m^2^), physical activity (MET-hours per week), histological type (small cell lung cancer, non-small lung cancer), AJCC stage (I–II, III–IV), comorbidities (yes, no), surgery (yes, no), chemotherapy (yes, no), radiotherapy (yes, no), targeted treatment (yes, no), and immunotherapy (yes, no).
Saira Amir, 2024	The CRIC Study	Cohort study	2003–2008	58	52.3	836	2,539	31.9	12 year	The National Cancer Institute Diet History Questionnaire (DHQ)	PDI hPDI uPDI	All mortality	Clinical site, age, sex, race, education, income, total energy intake, physical activity, smoking status, and alcohol use. Obesity status (categorical variable with cut off at 30 kg/m^2^), kidney function (estimated glomerular filtration rate), 24-hour urinary protein, diabetes, hypertension, history of cardiovascular disease, and use of angiotensin-converting enzyme inhibitors or angiotensin receptor blockers.
Zhilei Shan, 2023	NHS	Cohort study	1984–2020	50.2	100	31,263	75,230	/	36 year	Semi-quantitative food frequency questionnaire (FFQ)	hPDI	All mortality, Cardiovascular disease, Heart disease, Stroke, Cancer, Respiratory disease, Neurodegenerative disease	Multivariable analysis was adjusted for age, calendar year, race and ethnicity (NHS: Hispanic, non-Hispanic Black, non-Hispanic White, or other; HPFS, Black, White, or other), marriage status (married; divorced, separated, or single; or widowed), living status (alone or not alone), family history of myocardial infarction (yes or no), family history of diabetes (yes or no), family history of cancer (yes or no), menopausal status (pre- or postmenopausal [never, past, or current menopausal hormone use]; NHS only), multivitamin use (yes or no), aspirin use (yes or no), total energy intake (quintile), smoking status (never, former, or current smoker [1–14, 15–24, or ≥ 25 cigarettes/d]), alcohol drinking (0, 0.1–4.9, 5.0–14.9, 15.0–19.9, 20.0–29.9, or ≥ 30 g/d), physical activity (quintile), history of hypertension (yes or no), history of hypercholesterolemia (yes or no), and body mass index (<21, 21–24.9, 25–29.9, 30–34.9, or ≥ 35 [calculated as weight in kilograms divided by height in meters squared]).
	HPFS	Cohort study	1986–2020	53.3	0	22,900	44,085	/	34 year	Semi-quantitative food frequency questionnaire (FFQ)			
Jihye Kim, 2024	MEC	Cohort study	1993–2019	59	/	65,087	144,729	Men 26.6 Women 26.2	21.3 year	QFFQ	PDI hPDI uPDI	All mortality, CVD mortality, cancer mortality	Age at cohort entry, race and ethnicity, education, marital status, history of diabetes, body mass index, smoking status, pack-years of cigarette, physical activity, menopausal hormone therapy use for women only, alcohol consumption, and total energy intake

The extracted research topics, first authors, data sources, study types, study periods, demographic characteristics of participants (age, sex, quantity, and BMI), follow-up time, dietary assessment methods, study outcomes (types of mortality), adjusted HR or RR and the adjusted variables for each outcome were analysed.

**Table 2:** Subgroup analysis of plant-based diets and cardiovascular mortality, cancer mortality, and all-cause mortality.

Subgroup analysis of the relationship between of PDI, hPDI, uPDI and cancer mortality, CVD mortality and all mortality												
Subgroup	No. of studies	RR (95% CI)	*I*^2^%	*p* for heterogeneity	No. of studies	RR (95% CI)	*I*^2^%	*p* for heterogeneity	No. of studies	RR (95% CI)	*I*^2^%	*p* for heterogeneity
	PDI				hPDI				uPDI			
Cancer mortality	7	**0.88** (0.79, 0.98)	84.71%	0.02	8	**0.91** (0.83, 0.99)	85.61%	0.03	7	1.10 (0.97, 1.26)	93.11%	0.14
Location												
Korean	1	0.88 (0.75, 1.03)	–	–	1	1.00 (0.84, 1.19)	–	–	1	**1.23** (1.02, 1.48)	–	–
UK	1	1.04 (0.95, 1.13)	–	–	1	**0.90** (0.82, 0.99)	–	–	1	**1.16** (1.06, 1.27)	–	–
US	5	**0.85** (0.75, 0.95)	83.25%	<0.01	6	**0.90** (0.80, 1.00)	89.44%	<0.01	5	1.07 (0.90, 1.28)	95.38%	<0.01
Age												
≤55	2	0.78 (0.61, 1.01)	71.60%	0.06	3	**0.91** (0.86, 0.95)	0	0.52	2	**1.18** (1.02, 1.36)	0.00%	0.46
>55	5	0.91 (0.81, 1.03)	87.89%	<0.01	5	0.90 (0.78, 1.03)	91.55%	<0.01	5	1.09 (0.93, 1.27)	95.37%	<0.01
Total												
≤100,000	2	0.84 (0.56, 1.26)	87.46%	<0.01	2	0.98 (0.86, 1.12)	11.34%	0.29	2	1.01 (0.89, 1.14)	0.00%	0.37
>100,000	5	0.89 (0.79, 1.00)	87.11%	<0.01	6	**0.89** (0.81, 0.99)	89.06%	<0.01	5	1.13 (0.97, 1.32)	95.10%	<0.01
Female												
≤50%	2	**0.80** (0.69, 0.93)	84.27%	0.01	2	0.81 (0.56, 1.18)	–	–	2	1.15 (0.83, 1.60)	98.40%	<0.01
>50%	4	0.98 (0.91, 1.05)	32.72%	0.22	5	**0.93** (0.89, 0.97)	23.12%	0.27	4	1.08 (0.97, 1.19)	70.20%	0.02
Follow up time												
≤8	2	**0.73** (0.67, 0.80)	0.00%	0.48	2	**0.76** (0.58, 1.00)	80.87%	0.02	2	**1.26** (1.04, 1.54)	67.40%	0.08
>8	4	0.96 (0.87, 1.05)	76.02%	0.01	5	**0.94** (0.90, 0.98)	39.58%	0.16	4	**1.02** (0.94, 1.11)	71.74%	0.02
CVD mortality	7	**0.81** (0.76, 0.86)	0.00%	<0.01	9	**0.85** (0.77, 0.94)	85.13%	<0.01	8	**1.19** (1.07, 1.32)	80.03%	<0.01
Location												
Korean	1	0.77 (0.57, 1.04)	–	–	1	1.23 (0.90, 1.68)	–	–	1	**1.55** (1.07, 2.24)	–	–
Spain	–	–	–	–	1	**0.42** (0.20, 0.88)	–	–	1	1.11 (0.56, 2.19)	–	–
UK	1	**0.77** (0.66, 0.90)	–	–	1	0.92 (0.79, 1.08)	–	–	1	1.10 (0.93, 1.30)	–	–
US	5	**0.81** (0.75, 0.87)	63.50%	0.03	6	**0.83** (0.74, 0.92)	88.46%	<0.01	5	**1.18** (1.04, 1.34)	87.60%	<0.01
Age												
≤55	3	**0.75** (0.65, 0.85)	19.90%	0.29	5	0.94 (0.80, 1.10)	65.19%	0.02	4	1.22 (0.90, 1.65)	76.41%	0.01
>55	4	**0.83** (0.78, 0.88)	49.41%	0.12	4	**0.79** (0.72, 0.86)	73.70%	0.01	4	**1.19** (1.06, 1.34)	85.81%	<0.01
Total												
≤100,000	2	**0.75** (0.61, 0.92)	58.62%	0.12	3	0.83 (0.59, 1.16)	73.23%	0.02	3	1.13 (0.81, 1.59)	77.54%	0.01
>100,000	5	**0.82** (0.78, 0.87)	35.28%	0.19	5	**0.85** (0.76, 0.95)	89.16%	<0.01	5	**1.21** (1.08, 1.36)	82.83%	<0.01
Female												
≤50%	2	**0.81** (0.74, 0.88)	56.82%	0.13	2	**0.75** (0.65, 0.86)	83.96%	0.01	2	1.23 (0.95, 1.60)	95.10%	<0.01
>50%	4	**0.78** (0.68, 0.90)	67.89%	0.03	6	**0.88** (0.78, 0.99)	79.72%	<0.01	5	1.12 (0.97, 1.29)	64.72%	0.02
Follow up time												
≤8	2	**0.78** (0.72, 0.85)	0.00%	0.46	2	0.85 (0.55, 1.34)	91.15%	<0.01	2	**1.41** (1.29, 1.54)	0	0.96
>8	4	**0.81** (0.74, 0.88)	67.34%	0.03	6	**0.84** (0.76, 0.92)	80.38%	<0.01	5	**1.09** (1.00, 1.18)	58.74%	0.05
All mortality	13	**0.84** (0.79, 0.89)	81.99%	<0.01	15	**0.85** (0.80, 0.90)	89.83%	<0.01	14	**1.18** (1.09, 1.27)	89.97%	<0.01
Location												
German	1	**0.46** (0.29, 0.74)	–	–	1	0.76 (0.51, 1.14)	–	–	1	1.29 (0.84, 1.98)	–	–
China	2	0.94 (0.83, 1.07)	13.68%	0.28	2	**0.80** (0.73, 0.88)	5.63%	0.3	2	**1.18** (1.09, 1.26)	0.00%	0.47
Korean	1	**0.76** (0.68, 0.85)	–	–	1	1.05 (0.93, 1.18)	–	–	1	**1.30** (1.15, 1.47)	–	–
Spain	–	**–**	–	–	1	0.74 (0.52, 1.05)	–	–	1	1.18 (0.88, 1.58)	–	–
UK	1	**0.87** (0.81, 0.93)	–	–	1	**0.92** (0.86, 0.98)	–	–	1	**1.29** (1.20, 1.38)	–	–
US	8	**0.84** (0.78, 0.90)	85.09%	<0.01	9	**0.84** (0.78, 0.91)	93.00%	<0.01	8	**1.14** (1.03, 1.25)	93.55%	<0.01
Age												
≤55	4	**0.81** (0.73, 0.90)	69.13%	0.02	6	**0.90** (0.84, 0.96)	60.40%	0.04	5	1.17 (0.98, 1.39)	87.36%	<0.01
>55	9	**0.85** (0.80, 0.91)	83.58%	<0.01	9	**0.82** (0.75, 0.90)	93.33%	<0.01	9	**1.18** (1.08, 1.29)	91.80%	<0.01
Total												
≤100,000	8	**0.85** (0.75, 0.96)	80.60%	<0.01	9	**0.85** (0.81, 0.90)	23.04%	0.24	9	**1.14** (1.04, 1.26)	73.47%	<0.01
>100,000	5	**0.83** (0.77, 0.88)	86.70%	<0.01	6	**0.85** (0.78, 0.94)	96.67%	<0.01	5	**1.22** (1.08, 1.37)	95.92%	<0.01
Female												
≤50%	4	**0.79** (0.69, 0.91)	86.11%	<0.01	4	**0.74** (0.58, 0.94)	96.87%	<0.01	4	1.25 (0.97, 1.59)	96.30%	<0.01
>50%	8	**0.86** (0.81, 0.92)	78.18%	<0.01	10	**0.88** (0.85, 0.92)	59.46%	0.01	9	**1.14** (1.06, 1.22)	80.15%	<0.01
Follow up time												
≤8	5	**0.81** (0.71, 0.93)	87.07%	<0.01	5	**0.75** (0.64, 0.88)	90.24%	<0.01	5	**1.30** (1.17, 1.45)	74.80%	<0.01
>8	7	**0.87** (0.81, 0.92)	75.59%	<0.01	9	**0.87** (0.86, 0.89)	7.01%	0.38	8	**1.10** (1.02, 1.18)	84.24%	<0.01

Heterogeneity analysis was conducted on the basis of region, age, proportion of female participants, total number of participants, and follow-up duration. RR and 95% confidence intervals (CIs) were calculated. I^2^ ≤ 50% and I^2^ > 50% indicate significant and nonsignificant heterogeneity, respectively. Statistically significant RR values are bolded in the table.

### Cancer mortality

**Figure 2:**
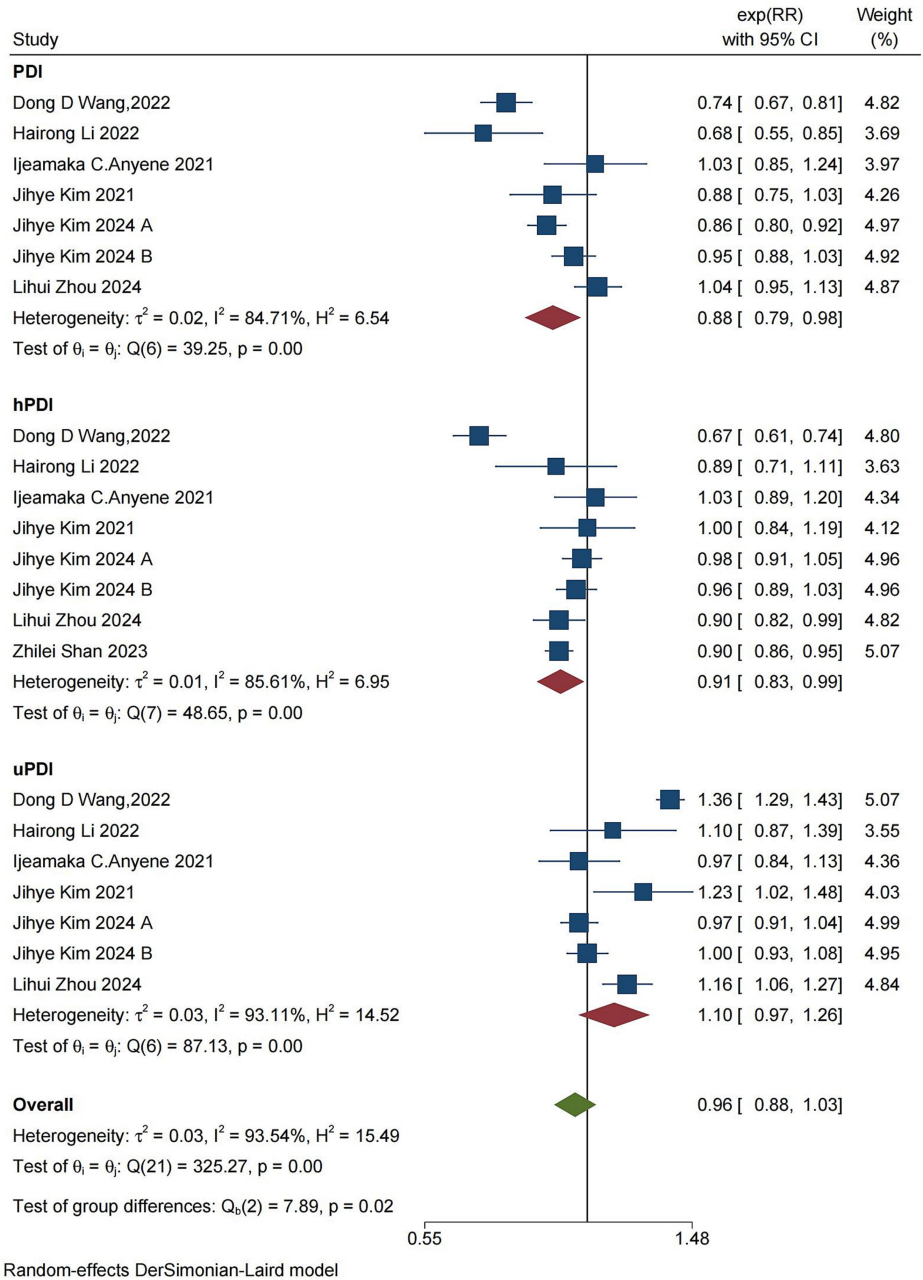
Forest plot of the summary analysis of cancer mortality rates and PDI, hPDI, and uPDI data. The forest plot displays the meta-analysis of cancer and PDI, hPDI, and uPDI. RR, relative risk; CI, confidence interval. The area of each square is inversely proportional to the variance of the estimated log RR (i.e., weight percentage), whereas the horizontal line represents the 95% CI for each individual study. The vertical axis of the red diamond represents the point estimate of the overall RR, with the horizontal axis representing the 95% CI. The vertical solid line indicates RR = 1.

Eight studies, reported across seven articles, investigated the associations between PDI, hPDI, uPDI, and cancer mortality (Figure 2). The pooled results indicated that PDI is associated with a reduced risk of cancer mortality (RR = 0.88, [95% CI 0.78–0.98], τ^2^: 0.02, I^2^: 84.71%) (Supplementary Figure S1). Furthermore, hPDI was negatively correlated with cancer mortality (RR = 0.91, [95% CI 0.83–0.99], τ^2^: 0.01, I^2^: 85.61%) (Supplementary Figure S2), albeit with high interstudy heterogeneity observed in the results. On the other hand, uPDI was associated with the risk of cancer mortality (RR = 1.10, [95% CI 0.97–1.26], τ^2^: 0.03, I^2^: 93.11%) (Supplementary Figure S3).

### CVD mortality

**Figure 3:**
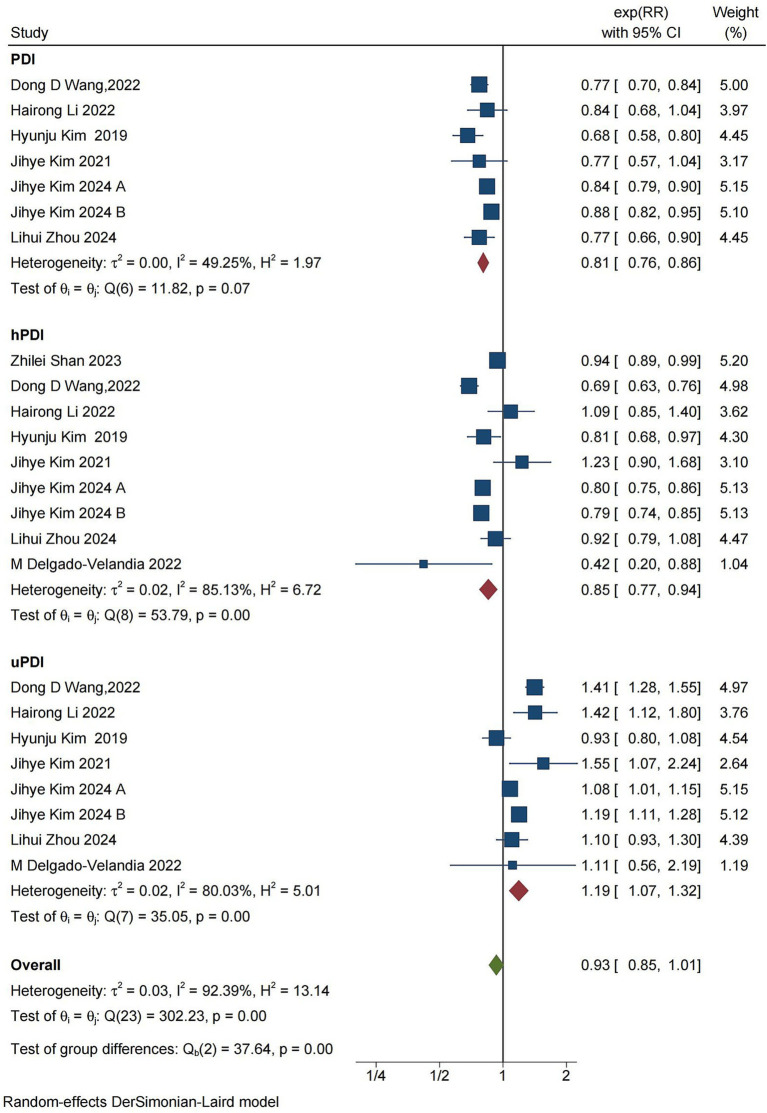
Forest plot of cardiovascular mortality and PDI, hPDI, and uPDI data classification and summary analysis. The forest plot displays the meta-analysis of cardiovascular disease and PDI, hPDI, and uPDI. RR, relative risk; CI, confidence interval. The area of each square is proportional to the inverse of the variance of the estimated log RR (i.e., weight percentage), whereas the horizontal line represents the 95% CI for each individual study. The vertical solid line indicates RR = 1. The red diamond on the y-axis represents the point estimate of the overall RR, with the x-axis representing the 95% CI.

Nine studies, reported across eight articles, investigated the associations between PDI, hPDI, uPDI, and cardiovascular mortality (Figure 3). The pooled results showed that PDI can reduce the risk of CVD mortality (RR = 0.81, [95% CI 0.76–0.86], τ^2^: 0.00, I^2^:49.25%) (Supplementary Figure S4), with low heterogeneity between studies. Furthermore, hPDI was negatively associated with CVD mortality (RR = 0.85, [95% CI 0.77–0.94], τ^2^: 0.02, I^2^: 85.13%) (Supplementary Figure S5), although there was high heterogeneity present in the results for this parameter. On the other hand, uPDI was associated with an increased risk of CVD mortality (RR = 1.19, [95% CI 1.07–1.32], τ^2^: 0.02, I^2^: 80.03%) (Supplementary Figure S6), with high heterogeneity observed across the studies.

### All mortality

**Figure 4:**
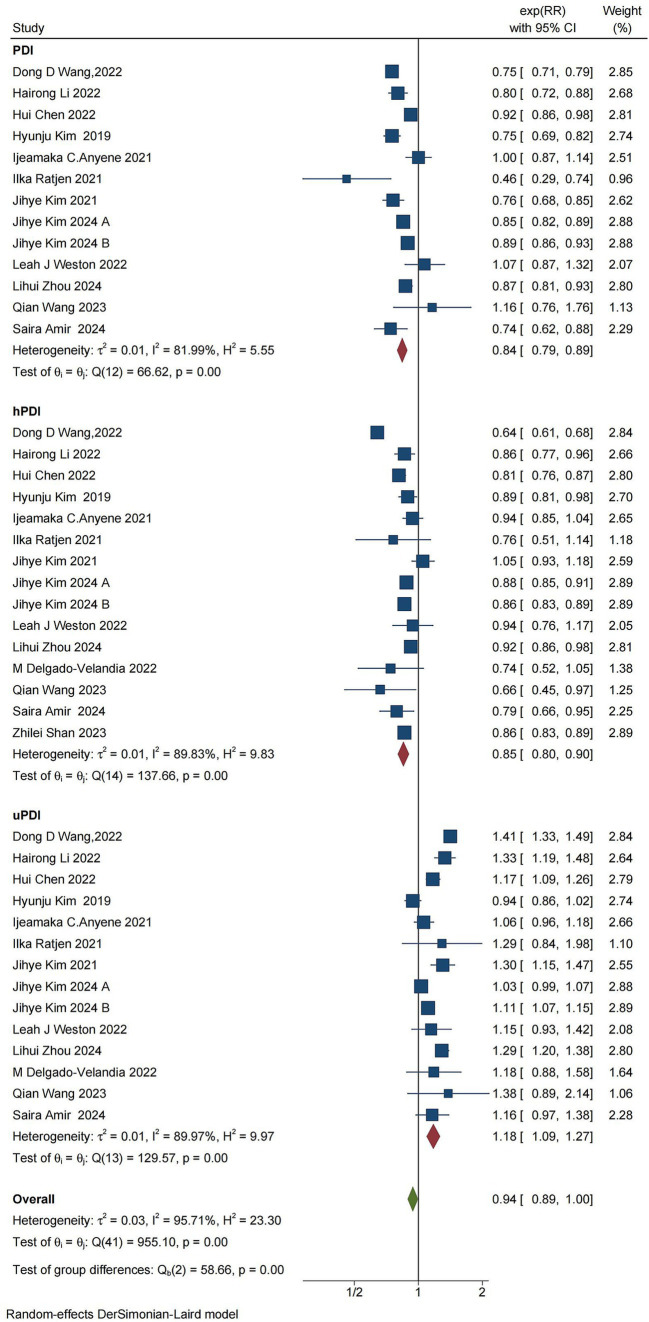
Forest plot for the meta-analysis of overall mortality and PDI, hPDI, and uPDI. The forest plot displays the meta-analysis of overall mortality and PDI, hPDI, and uPDI. RR, relative risk; CI, confidence interval. The area of each square is proportional to the reciprocal of the variance of the estimated log RR (i.e., weight percentage), whereas the horizontal line represents the 95% CI for each individual study. The vertical solid line indicates RR = 1. The red diamond on the y-axis represents the point estimate of the overall RR, with the x-axis representing the 95% CI.

The combined findings from 15 studies, reported in 14 articles, on PDI, hPDI, uPDI, and all-cause mortality indicated that PDI was associated with a reduction in overall mortality (RR = 0.84, [95% CI 0.79–0.89], τ^2^:0.01, I^2^: 81.99%) (Figure 4). There was substantial heterogeneity among the studies. Sensitivity analysis revealed no conflicting results, indicating a significant negative correlation between the PDI score and overall mortality (Supplementary Figure S7). Similarly, hPDI was associated with a reduction in overall mortality (RR = 0.85, [95% CI 0.80–0.90], τ^2^: 0.01, I^2^: 89.83%) (Supplementary Figure S8), with high heterogeneity among the studies as well as no contradictory results from the sensitivity analysis, suggesting a significant negative correlation between hPDI and all-cause mortality. On the other hand, uPDI resulted in an increased risk of overall mortality (RR = 1.18, [95% CI 1.09–1.27], τ^2^:0.01, I^2^:89.97%) (Supplementary Figure S9), with high heterogeneity between studies and no conflicting results from the sensitivity analysis, indicating a significant positive association between uPDI and all-cause mortality.

### Subgroup analysis

To further assessed the correlation between covariate factors and the results, we conducted a stratified analysis of the results on the basis of country, region, age proportion of the female population, and follow-up time. Detailed information is shown in Table 2.

In the hPDI analysis, most studies originated from the United States, with fewer contributions from other regions. Future research should focus on increasing studies from diverse regions to provide more comprehensive data. Subgroup analysis by age revealed significant differences across all three mortality outcomes. High levels of hPDI were not associated with cancer death in individuals older than 55 years (RR_>55_ = 0.90 [95% CI 0.78–1.03]), whereas high levels of hPDI were not associated with cardiovascular death in those aged 55 years or younger (RR_≤55_ = 0.94 [95% CI 0.80–1.10]). The protective effect of high levels of hPDI on all-cause death was more pronounced in individuals older than 55 years (RR_≤55_ = 0.90 [95% CI 0.84–0.96], RR_>55_ = 0.82 [95% CI 0.75–0.90]). Importantly, sample size may impact study accuracy; for populations ≤100,000, the protective effect of high hPDI on cancer death and CVD is not evident, and the difference is not statistically significant. The sex ratio had a greater impact on deaths from different factors; there was a consistent trend showing that the protective effect of high levels of hRDI was slightly reduced when analysing populations where women comprised more than 50% of the population (cancer mortality: RR_female_ > 50% = 0.93 [95% CI 0.89–0.97]; CVD mortality: RR_female_ > 50% = 0.88 [95% CI 0.78–0.99]. All mortality: RR_female_ > 50% = 0.88 [95% CI 0.85–0.92]). Finally, stratified analysis based on follow-up time suggested that follow-up duration may be a source of heterogeneity: analyses with more than 8 years of follow-up exhibited significantly lower heterogeneity and higher pooled estimates than those with shorter follow-up periods, while also demonstrating narrower confidence intervals and more stable outcomes in cancer mortality and all-cause mortality analyses.

In the analysis of uPDI, the impact of uPDI on cancer mortality was found to be minimal, with statistically significant differences observed in only a few cases. Significant differences were observed across the age subgroups for all three mortality outcomes, and there was a significant association between uPDI and the risk of cancer mortality in individuals aged ≤55 years (RR = 1.18, [95% CI 1.02–1.36]). Conversely, uPDI had a more pronounced effect on the risk of cardiovascular disease mortality (RR = 1.19, [95% CI 1.06–1.34]) and all-cause mortality (RR = 1.18, [95% CI 1.08–1.29]) in those aged >55 years. The statistical results indicated that sample size did not have a significant effect on the findings of the study. Subgroup analysis on the basis of the uPDI sex ratio revealed that adverse effects of uPDI were more prominent in analyses where the risk of all-cause mortality was greater than 50% female (RR = 1.14, [95% CI 1.06–1.22]). Additionally, a shorter follow-up time (≤8 years) intensified the adverse effects of uPDI on cancer, cardiovascular disease, and all-cause mortality, with an aggregate value of RR = 1.26 [95% CI 1.04–1.54], RR = 1.41[95% CI 1.29–1.54], RR = 1.30 [95% CI 1.17–1.45].

### Sensitivity analysis

Sensitivity analysis was carried out on all the results, and the results were stable with no contradictory results.

### Publication bias analysis

**Figure 5:**
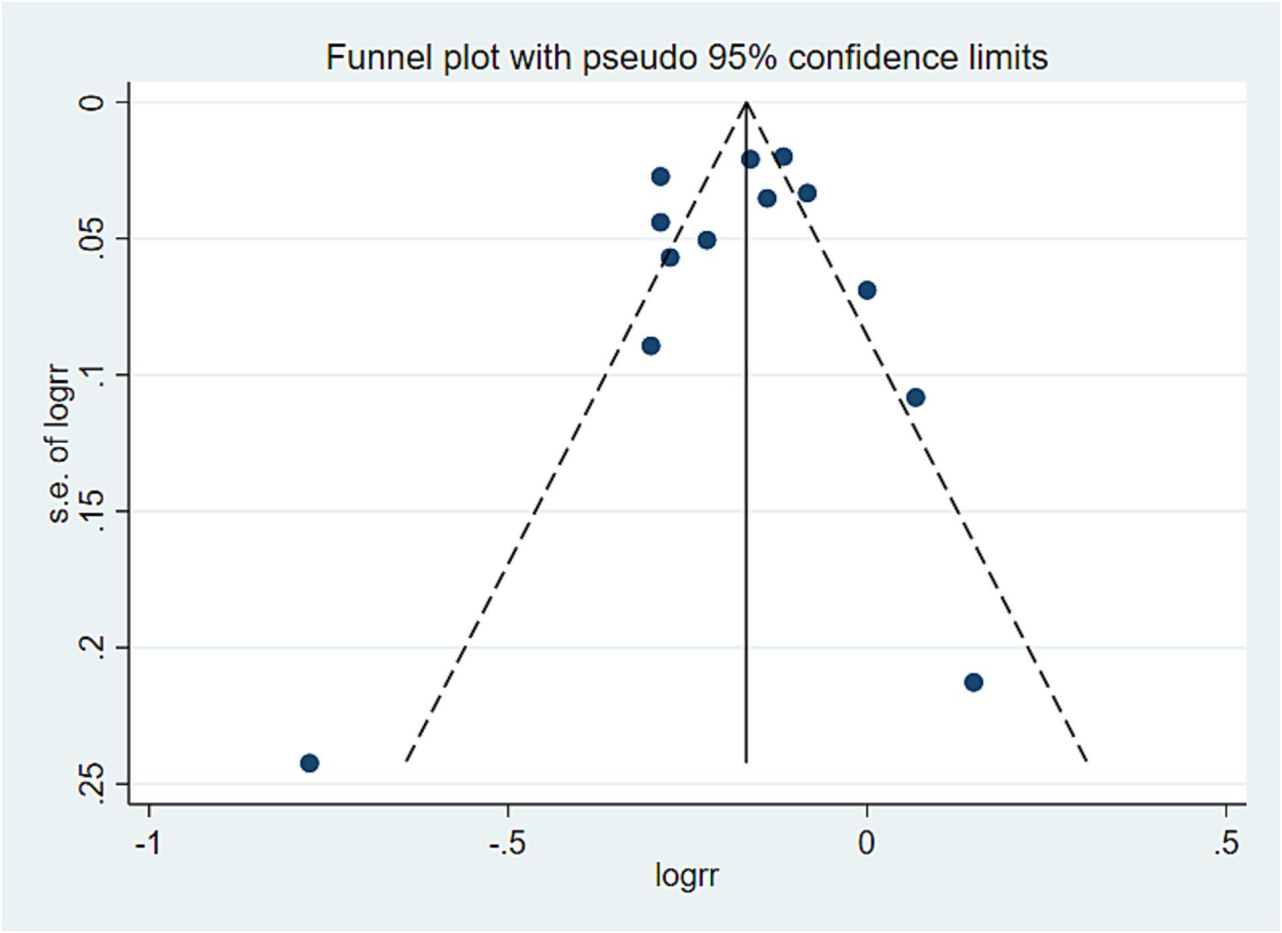
PDI and all-cause mortality offset analysis data. Visual observation was used to identify the presence of publication bias. If the funnel plot shows that the majority of studies are located at the top of the “funnel” with fewer at the base and that there is approximate symmetry on both sides, it suggests that publication bias is not significant. Conversely, if there is an obvious asymmetry, it indicates a clear publication bias.

**Figure 6:**
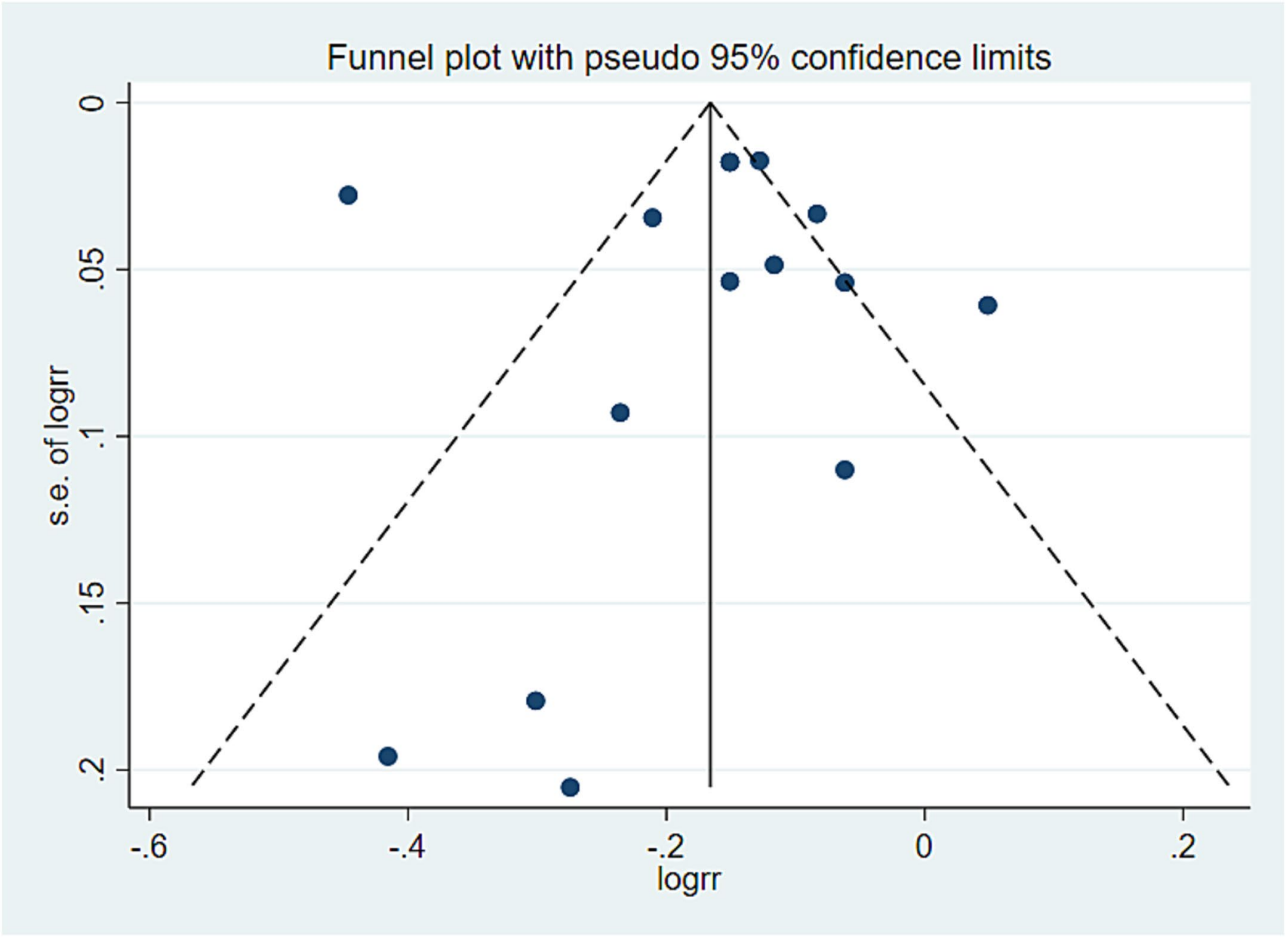
hPDI and all-cause mortality offset analysis data. Visual observation was used to identify the presence of publication bias. If the funnel plot shows that the majority of studies are located at the top of the “funnel” with fewer at the base and that there is approximate symmetry on both sides, it suggests that publication bias is not significant. Conversely, if there is an obvious asymmetry, it indicates a clear publication bias.

**Figure 7:**
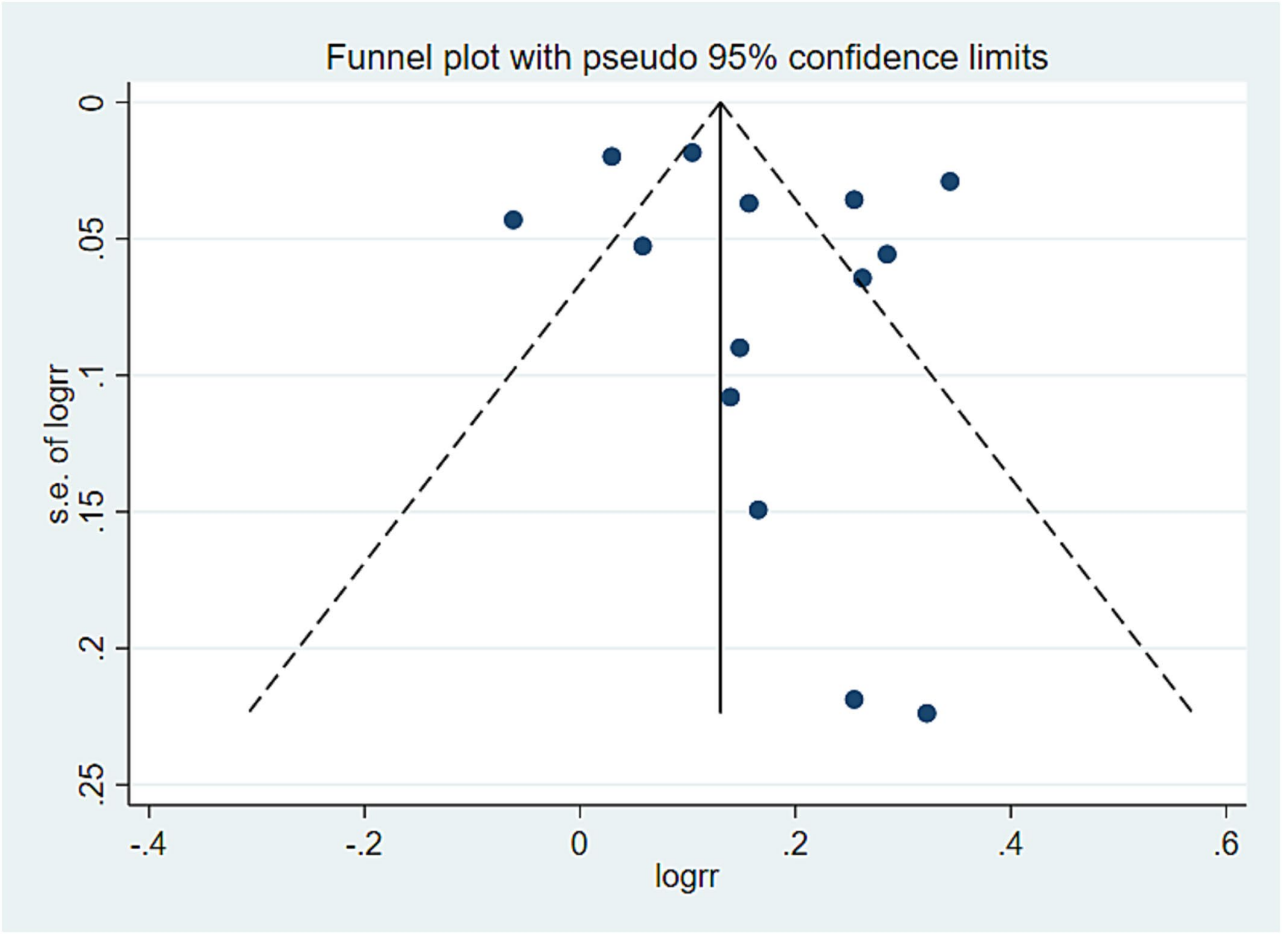
All-cause mortality offset analysis data. Visual observation was used to identify the presence of publication bias. If the funnel plot shows that the majority of studies are located at the top of the “funnel” with fewer at the base and that there is approximate symmetry on both sides, it suggests that publication bias is not significant. Conversely, if there is an obvious asymmetry, it indicates a clear publication bias.

In this study, a publication bias analysis was conducted on the results of 10 or more included studies. The analysis revealed that in the association analysis between the PDI score and overall risk of death, the data distribution in Figure 5 was approximately symmetrical and relatively concentrated. Egger test results (*p* = 0.720 > 0.05) indicated no significant bias. In the association analysis between hPDI and the overall risk of death, the data distribution in Figure 6 was also approximately symmetrical and relatively concentrated. Egger test results (*p* = 0.931 > 0.05) indicated no significant bias. Similarly, in the association analysis between uPDI and overall risk of death, the data distribution in Figure 7 was approximately symmetrical and relatively concentrated, with an Egger test result (*p* = 0.398 > 0.05), indicating that no significant bias was present. In this study, no significant deviation was found in the funnel plot results of the cancer and cardiovascular bias analyses, as detailed in the attachment.

## Discussion

Adherence to the hPDI is a dietary measure that emphasizes increased consumption of nutritious plant-based foods, such as whole grains, vegetables, nuts, legumes, coffee, and tea (21). Researches have demonstrated that plant-based diets are associated with significantly lower modifiable risk factors, including body mass index (BMI), blood glucose levels, inflammation, blood pressure, cardiometabolic risk status, reduced risk of nonalcoholic fatty liver disease (NAFLD), and decreased liver fat content (21, 34–38). Our analysis also revealed that high adherence to a plant-based diet has a substantial preventive effect on all-cause mortality, cardiovascular mortality, and cancer mortality. Furthermore, this preventive effect might be more pronounced in individuals over 55 years old. These findings were consistent with those of previous studies (3). As individuals age, changes in heart function, such as increased blood pressure and the onset of arrhythmias, may occur, elevating cardiovascular risk (39). Moreover, studies have indicated that older individuals are at a higher risk of developing cancer (40). A plant-based diet contains many components known to have beneficial effects on inflammation, cellular redox, metabolic processes, and endothelial function. Some studies have shown that certain components of a healthy plant-based diet have anticancer effects (41). Additionally, dietary fiber in a plant-based diet can reduce levels of inflammatory markers (42, 43), which may enhance the responsiveness of older individuals to the benefits of a healthy plant-based diet. Interestingly, in the subgroup analysis based on the sex ratio, compliance with hPDI might have a more significant protective effect on men. Compared with women, men generally tended to engage in more intense work and exercise and prefer diets high in fat and meat. These factors could lead to an increase in cardiovascular and cellular inflammation. Adherence to hPDI results in a diet rich in dietary fiber, antioxidants, unsaturated fats, and micronutrients, while being low in saturated fats and heme iron. This dietary pattern can enhance human metabolism, thereby reducing the risk of coronary heart disease (44), which may explain the differences observed in the protective effects of hPDI between sexes.

uPDI is a dietary index for a group with a less healthy intake of plant-based foods and an animal-based diet (19). Previous studies have shown that uPDI is associated with a greater risk of NAFLD and intrahepatic steatosis (21), and other studies have shown that uPDI is linked to an increased risk of chronic disease and mortality. In this study, we observed a significant positive correlation between the use of uPDI and cardiovascular mortality as well as all-cause mortality. This correlation may be more pronounced in individuals over the age of 55. Overweight and obesity currently represent the fifth leading cause of death globally, with obesity contributing to increased prevalence, morbidity, and premature mortality of associated diseases (45). Research has indicated that obesity leads to changes in the structure and function of the heart; alterations in heart muscle structure increase the risk of atrial fibrillation and sudden cardiac death (46). Additionally, uPDI contributes to a high glycemic index and load, decreased fibre content, low micronutrient content, and high caloric content (47), which may promote weight gain. For older individuals, vascular endothelial function decreases while inflammation levels increase; This weakening vascular elasticity further increases the risk of CVD, among other conditions. Furthermore, our analysis revealed another noteworthy result: in a subgroup analysis based on follow-up time, high-level study results demonstrated that uPDI had a more significant adverse effect on participants followed for less than 8 years. This might be attributed to the fact that an unhealthy plant-based diet has a substantial short-term impact on the human body. This could be related to the strong link between the gut microbiota and metabolic health (48). High-fat intake may promote hyperorexia by increasing mucosal permeability and toll-like receptor activation, which stimulates innate immune responses (inflammasome), leading to alterations in the gut microbiome composition (49). Short-term plant-based dietary patterns may be sufficient to modify gut microbiome diversity (50). Additionally, higher levels of added sugars in an unhealthy plant-based diet may also contribute to increased energy intake through neurochemical changes in the brain (51). Multiple animal studies have demonstrated that a high-fat intake can disrupt energy balance by providing excess energy, leading to hypothalamic damage and impairing the hypothalamus’s normal functions of nutritional sensing and energy regulation (52, 53). These disruptions may cause changes in disease incidence and mortality over a short period. Such mechanisms could explain the pronounced short-term effects of uPDI on the human body.

### Actual impact

This study aimed to systematically analyze all available research assessing the associations between plant-based diets and all-cause mortality. A total of 14 articles were included in the analysis, comprising 15 studies on overall mortality risk, 9 studies on cardiovascular mortality, and 8 studies on cancer mortality. These results suggest a significant association between plant-based diets and reduced all-cause mortality, indicating that adherence to a healthy plant-based diet lowers the risk of death.

### Advantage

This study represented the first meta-analysis examining all-cause mortality, cancer mortality, and CVD mortality using standardized plant-based diet scores. The analysis investigated the associations between PDI, hPDI, and uPDI and the three types of death. By integrating the analysis of plant-based diets, this study aimed to reduce heterogeneity resulting from differences in dietary patterns. All included studies were cohort studies with high-quality literature, ensuring the reliability of the analysis results. These findings effectively confirmed a correlation between plant-based dietary patterns and various health outcomes. Subgroup analysis was utilized for pooled analyses with high heterogeneity to identify sources of partial heterogeneity, such as duration of follow-up, age, region, proportion of female participants, and total number of study cases.

### Limitations

The review in this study was systematic and exhaustive, drawing conclusions about the various causes of death and different plant-based diets. The studies included a total of 976,396 participants, providing a large sample size that offered evidence to detect a statistically significant relationship between plant-based diets and all-cause mortality. However, there are several limitations to this analysis. First, while the current meta-analysis involves a sufficient sample size for the overall analysis, the occurrence of diseases is closely related to region and age. Currently, there are too few original articles involving relevant factors such as region and average age, leading to some bias in the results. In the future, it is necessary to increase the research of multi-region and multi-species. Second, there may be small study effects that threaten the validity of the meta-analysis results. We used various methods to evaluate publication bias (the main cause of the small-scale study effect), including visually assessing funnel plot asymmetry and conducting Egger regression tests (*p* = 0.000 < 0.05), indicating that possible publication bias exists. Therefore, we further processed the asymmetric funnel plot via the shear compensation method, which revealed that our analysis was stable and not affected by publication bias. Owing to the limited number of included studies, publication bias has little significance in some analyses; however, additional relevant studies are needed to address this issue comprehensively. Finally, due to the limited availability of subgroup data, further sources of heterogeneity could not be identified, such as pooled analyses comparing cancer mortality with CVD mortality. Additionally, owing to the relative lack of data from studies involving different levels of exposure to plant-based diets, we were unable to perform dose–response analyses to obtain more detailed results.

## Conclusion

A plant-based diet plays a crucial role in promoting public health by reducing the risk of chronic diseases and premature death. The findings from the data analysis indicated that adhering to a high-level healthy plant-based diet is linked to lower mortality rates, whereas greater adherence to an unhealthy plant-based diet is associated with higher mortality rates. Therefore, it was important to prioritize healthy plant-based foods and limit the consumption of less healthy plant-based foods and certain animal-based foods to achieve significant health benefits.

## Data Availability

The original contributions presented in the study are included in the article/Supplementary material, further inquiries can be directed to the corresponding authors.

## Supplementary material

Supplementary TABLE S1 Literature search strategy - displaying literature search terms.

Supplementary TABLE S2 Newcastle - ottawa quality assessment scale.

Supplementary Figure S1 Presents the data on the correlation between PDI and Cancer mortality.

Supplementary Figure S2 Presents the data on the correlation between hPDI and Cancer mortality.

Supplementary Figure S3 Presents the data on the correlation between uPDI and Cancer mortality.

Supplementary Figure S4 Presents the data on the correlation between PDI and CVD mortality.

Supplementary Figure S5 Presents the data on the correlation between hPDI and CVD mortality.

Supplementary Figure S6 Presents the data on the correlation between uPDI and CVD mortality.

Supplementary Figure S7 Presents the data on the correlation between PDI and all mortality.

Supplementary Figure S8 Presents the data on the correlation between hPDI and all mortality.

Supplementary Figure S9 Presents the data on the correlation between uPDI and all mortality.
