# Supplementary material for: Plant-based diet and risk of all-cause mortality: a systematic review and meta-analysis
Source: Front Nutr. 2024 Oct 23;11:1481363. doi: 10.3389/fnut.2024.1481363 (PMC11537864; doi:10.3389/fnut.2024.1481363)

**Supplemental Table S1**: Literature Search Strategy - Displaying Literature Search Terms

**Supplemental Table S2**: NEWCASTLE - OTTAWA QUALITY ASSESSMENT SCALE

**Supplementary Figure S1:** presents the data on the correlation between PDI and Cancer mortality.

**Supplementary Figure S2:** presents the data on the correlation between hPDI and Cancer mortality.

**Supplementary Figure S3:** presents the data on the correlation between uPDI and Cancer mortality.

**Supplementary Figure S4:** presents the data on the correlation between PDI and CVD mortality.

**Supplementary Figure S5:** presents the data on the correlation between hPDI and CVD mortality.

**Supplementary Figure S6:** presents the data on the correlation between uPDI and CVD mortality.

**Supplementary Figure S7:** presents the data on the correlation between PDI and All mortality.

**Supplementary Figure S8:** presents the data on the correlation between hPDI and All mortality.

**Supplementary Figure S9:** presents the data on the correlation between uPDI and All mortality.

**Supplemental Table S1**: Medical subject headings (MeSH) and non-MeSH terms used to search relevant publications on the relation between plant-based diets and all cause mortality ^1^

| Database | Step | Terms | Results |
| --- | --- | --- | --- |
| PubMed | 1 | ("Diet, Plant-Based" [Mesh] OR "Diet, Vegetarian"[Mesh]) OR ("Diet, Plant-Based"[Title/Abstract] OR "Diet, Vegetarian"[Title/Abstract] OR "Diet, Plant Based"[Title/Abstract] OR "Diets, Plant-Based"[Title/Abstract] OR "Plant-Based Diets"[Title/Abstract] OR "Plant-Based Diet"[Title/Abstract] OR "Plant Based Diet"[Title/Abstract] OR "Plant-Based Nutrition"[Title/Abstract] OR "Nutrition, Plant-Based"[Title/Abstract] OR "Plant Based Nutrition"[Title/Abstract] OR "vegetarian"[Title/Abstract] OR "vegan"[Title/Abstract] OR "vegetable"[Title/Abstract] OR "dietary fiber"[Title/Abstract] OR "dietary pattern"[Title/Abstract] OR "food pattern"[Title/Abstract]) | 67266 |
|  | 2 | "mortality"[Mesh] OR "mortality"[Title/Abstract] OR "mortality rate"[Title/Abstract] OR "death"[Title/Abstract] OR "death rate"[Title/Abstract] | 2026806 |
|  | 3 | #1 AND #2 | **3548** |
| Web of Science | 1 | TS=("Diet, Plant-Based" OR "Diet, Vegetarian" OR "Diet, Plant Based" OR "Diets, Plant-Based" OR "Plant-Based Diets" OR "Plant-Based Diet" OR "Plant Based Diet" OR "Plant-Based Nutrition" OR "Nutrition, Plant-Based" OR "Plant Based Nutrition" OR "vegetarian" OR "vegan" OR "vegetable" OR "dietary fiber" OR "dietary pattern" OR "food pattern") | 252737 |
|  | 2 | TS=（"mortality" OR "mortality rate"OR "death" OR "cardiovascular disease" OR "death rate" ） | 2156764 |
|  | 3 | #1 AND #2 | **15235** |
| Embase | 1 | ('plant-based diet'/exp OR 'vegetarian diet'/exp) OR ('diet, plant-based':ti,ab,kw OR 'diet, vegetarian':ti,ab,kw OR 'diet, plant based':ti,ab,kw OR 'diets, plant-based':ti,ab,kw OR 'plant-based diets':ti,ab,kw OR 'plant-based diet':ti,ab,kw OR 'plant based diet':ti,ab,kw OR 'plant-based nutrition':ti,ab,kw OR 'nutrition, plant-based':ti,ab,kw OR 'plant based nutrition':ti,ab,kw OR 'vegetarian':ti,ab,kw OR 'vegan':ti,ab,kw OR 'vegetable':ti,ab,kw OR 'dietary fiber':ti,ab,kw OR 'dietary pattern':ti,ab,kw OR 'food pattern':ti,ab,kw) | 88181 |
|  | 2 | 'mortality'/exp OR 'mortality':ti,ab,kw OR 'mortality rate':ti,ab,kw OR 'death':ti,ab,kw OR 'death rate':ti,ab,kw | 3047199 |
|  | 3 | #1 AND #2 | **6193** |

^1^ Two investigators searched the online databases independently.

| Author, year | Selection | | | | Comparability | Outcome | | | Overall  quality |
| --- | --- | --- | --- | --- | --- | --- | --- | --- | --- |
|  | Representative of cohort | Selection of cohort | Exposure ascertainment | No history of disease | Comparability of cohorts | Outcome assessment | Follow-up long enough (median ≥ 5 years) | Adequacy of follow up |  |
| Dong D Wang 2022 | 0 | 1 | 1 | 1 | 2 | 1 | 0 | 1 | 7 |
| Hairong Li 2022 | 1 | 1 | 1 | 1 | 2 | 1 | 1 | 1 | 9 |
| Hui Chen 2022 | 1 | 1 | 1 | 1 | 1 | 1 | 1 | 1 | 8 |
| Hyunju Kim 2019 | 1 | 1 | 1 | 1 | 1 | 1 | 1 | 1 | 8 |
| Ijeamaka C.Anyene 2021 | 1 | 1 | 1 | 1 | 2 | 1 | 1 | 1 | 9 |
| Ilka Ratjen 2021 | 1 | 1 | 1 | 1 | 1 | 1 | 1 | 1 | 8 |
| Jihye Kim 2021 | 1 | 1 | 1 | 1 | 1 | 1 | 0 | 1 | 7 |
| Leah J Weston 2022 | 1 | 1 | 1 | 1 | 2 | 1 | 1 | 1 | 9 |
| Lihui Zhou 2024 | 1 | 1 | 1 | 1 | 2 | 1 | 1 | 1 | 9 |
| M Delgado-Velandia 2022 | 1 | 1 | 1 | 1 | 2 | 1 | 1 | 1 | 9 |
| Qian Wang 2023 | 1 | 1 | 1 | 1 | 2 | 1 | 0 | 0 | 7 |
| Saira Amir 2024 | 1 | 1 | 1 | 1 | 2 | 1 | 1 | 1 | 9 |
| Zhilei Shan 2023 | 0 | 1 | 1 | 1 | 2 | 1 | 1 | 1 | 8 |
| Jihye Kim 2024 | 1 | 1 | 1 | 1 | 2 | 1 | 1 | 1 | 9 |

**Supplemental Table S2**:**NEWCASTLE - OTTAWA QUALITY ASSESSMENT SCALE (COHORT STUDIES)**

**Supplementary Figure S1:** presents the data on the correlation between PDI and Cancer mortality.
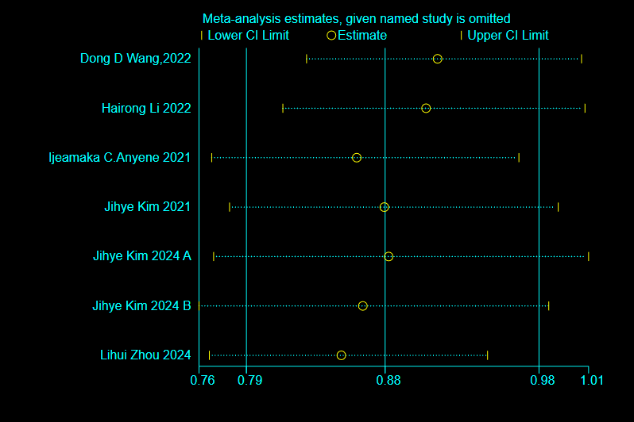

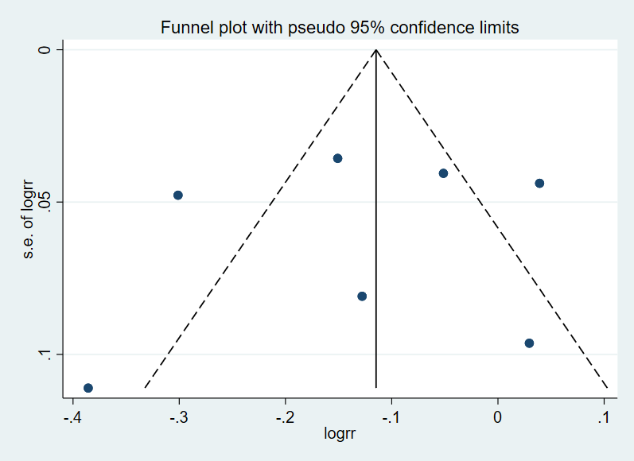

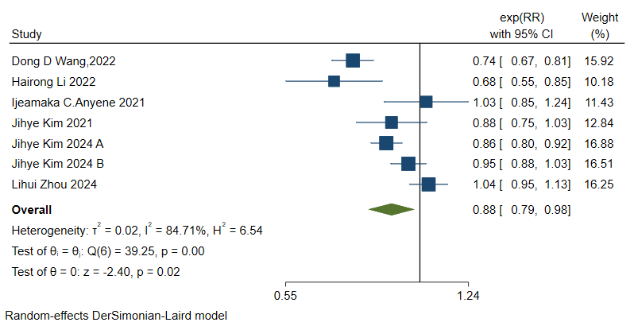


**Supplementary Figure S2:** presents the data on the correlation between hPDI and Cancer mortality.


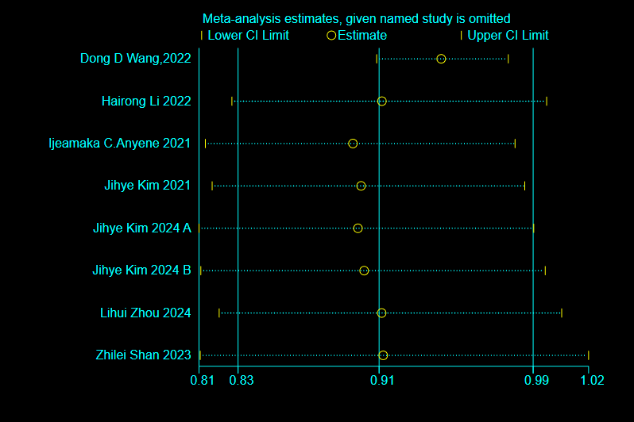

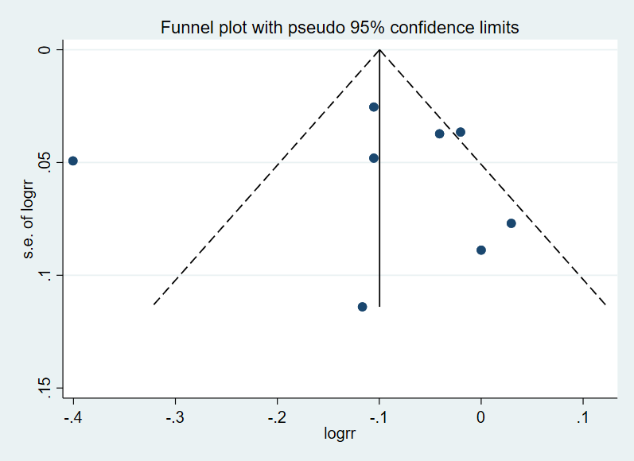

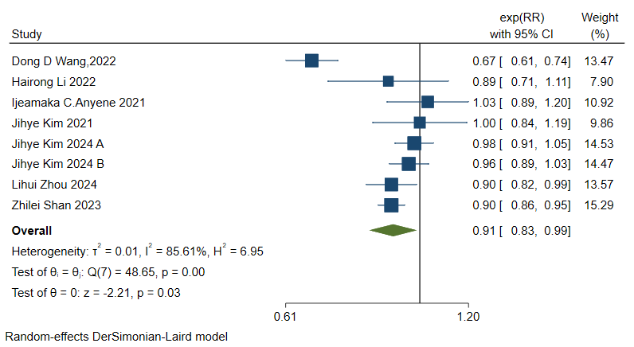


**Supplementary Figure S3:** presents the data on the correlation between uPDI and Cancer mortality.
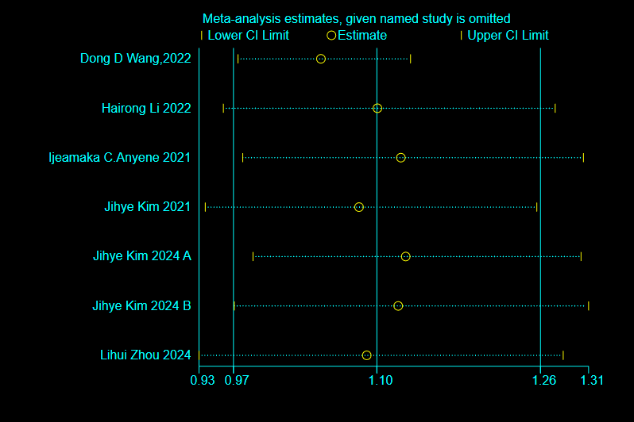

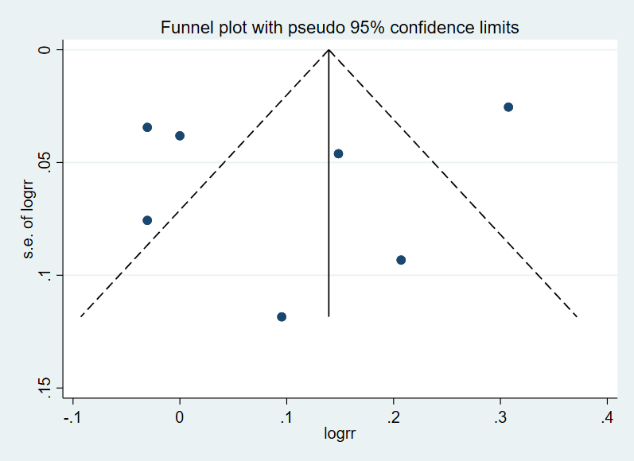

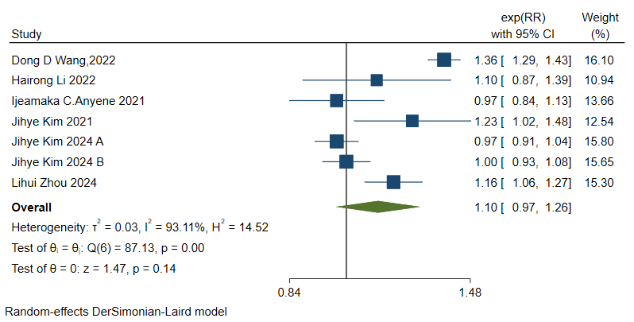


**Supplementary Figure S4:** presents the data on the correlation between PDI and CVD mortality.
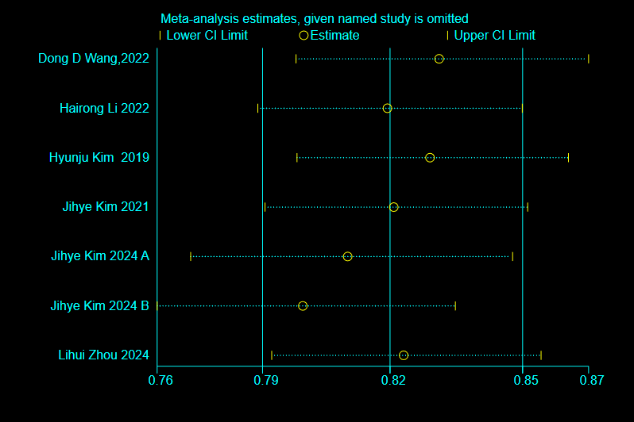

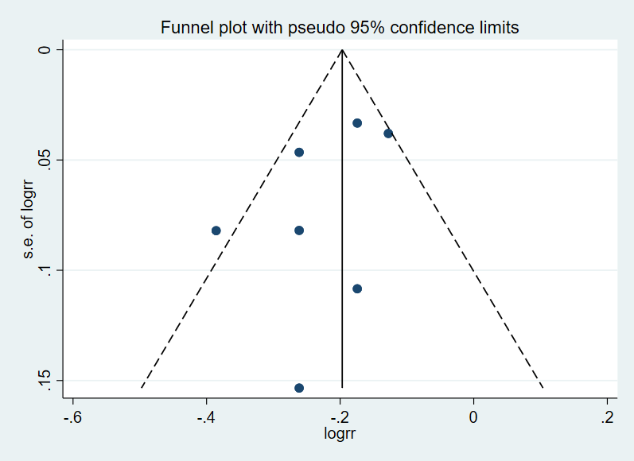

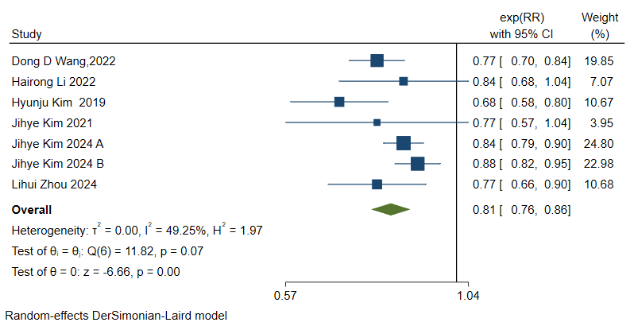


**Supplementary Figure S5:** presents the data on the correlation between hPDI and CVD mortality.


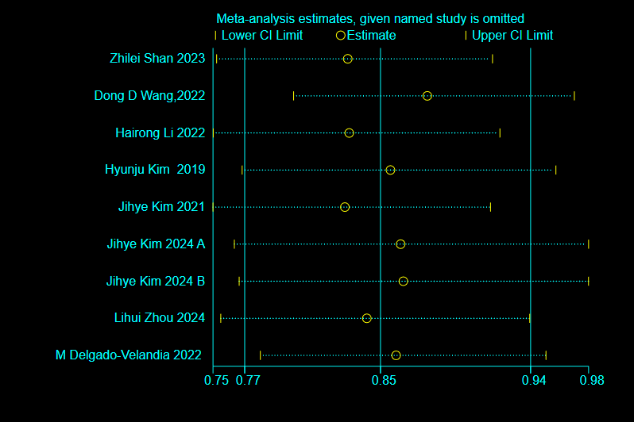

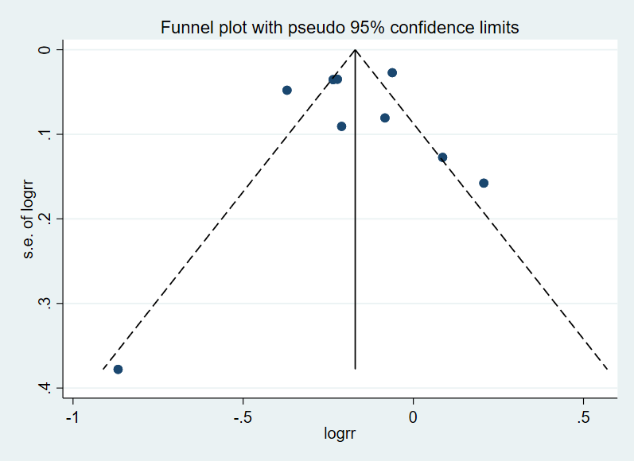

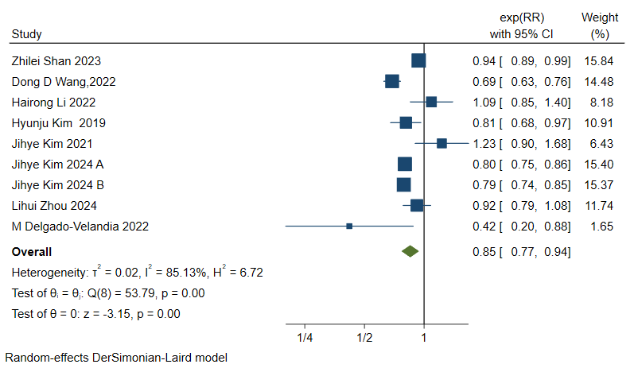


**Supplementary Figure S6:** presents the data on the correlation between uPDI and CVD mortality.


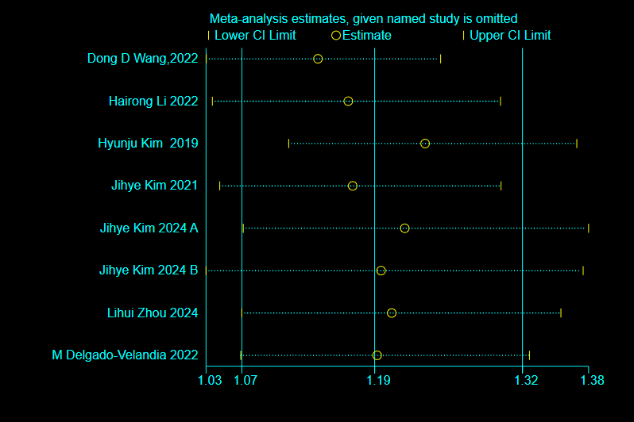

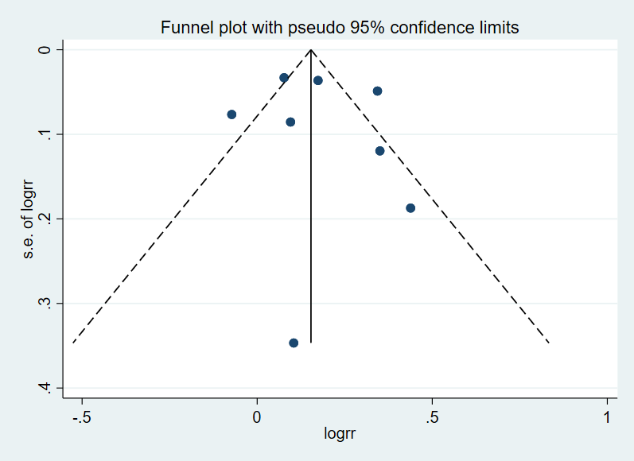

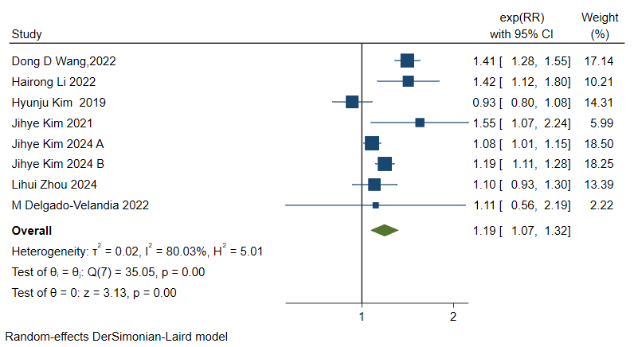


**Supplementary Figure S7:** presents the data on the correlation between PDI and All mortality.
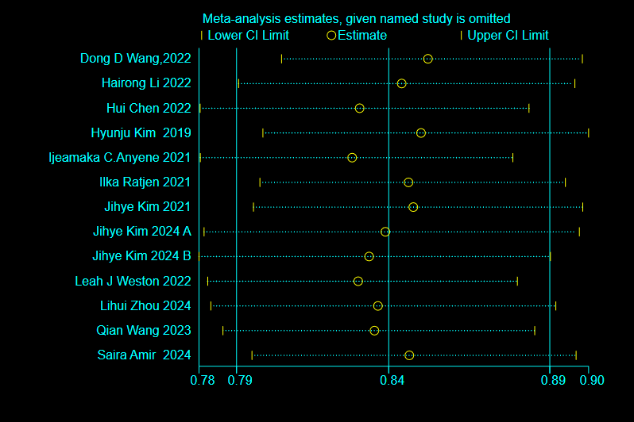

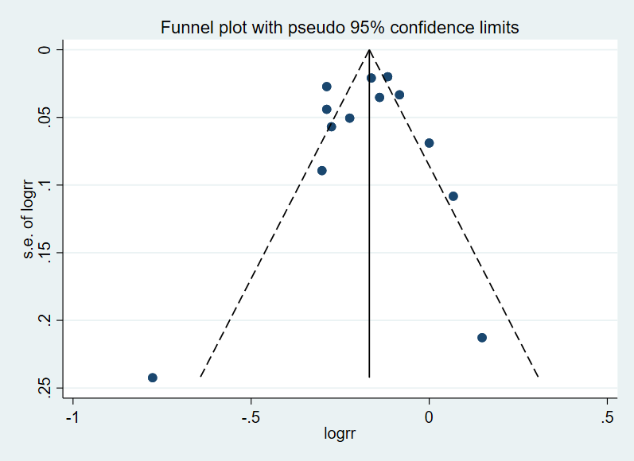

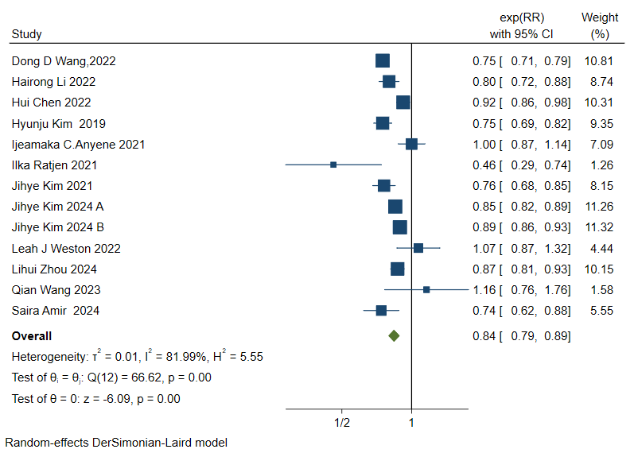


**Supplementary Figure S8:** presents the data on the correlation between hPDI and All mortality.


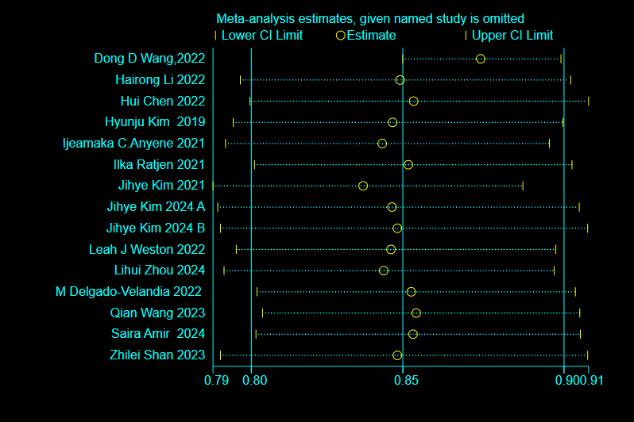

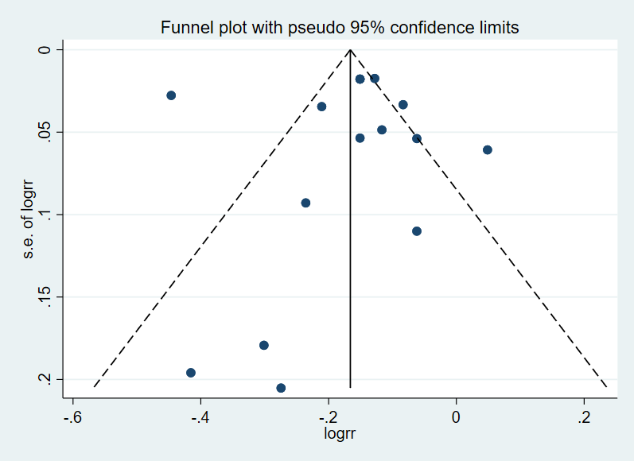

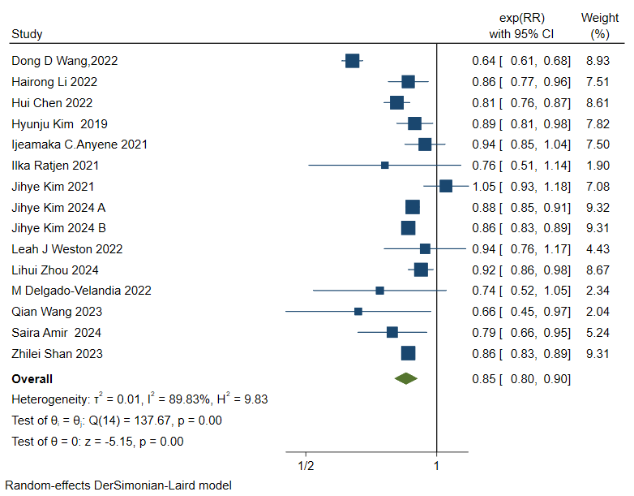


**Supplementary Figure S9:** presents the data on the correlation between uPDI and All mortality.


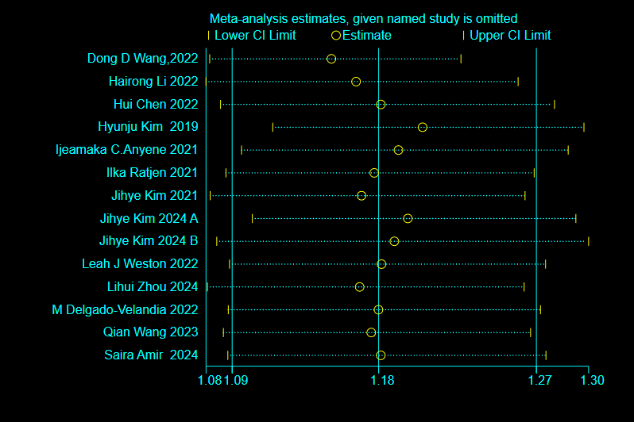

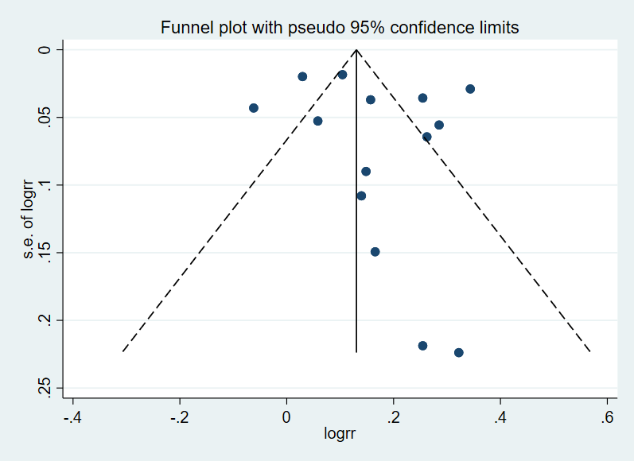

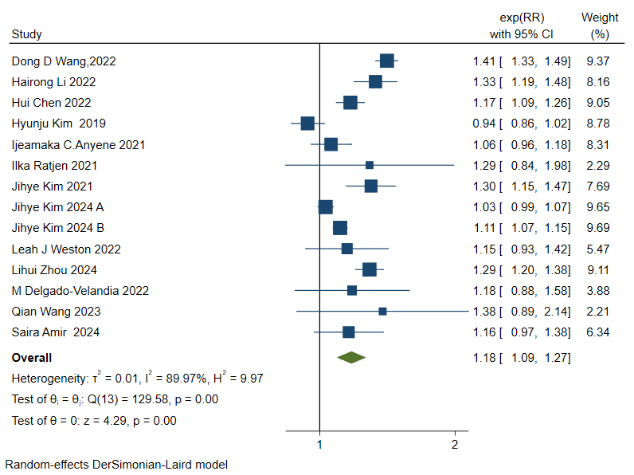

Supplement: Supplementary TABLE S1 — Literature search strategy - displaying literature search terms. [file Data_Sheet_1.docx]
